# Infectious Causes of Neoplasia in the Domestic Cat

**DOI:** 10.3390/vetsci9090467

**Published:** 2022-08-30

**Authors:** Kerry E. Rolph, Ryan P. Cavanaugh

**Affiliations:** Center for Integrative Mammalian Research, Ross University School of Veterinary Medicine, P.O. Box 334, Basseterre, St. Kitts, West Indies

**Keywords:** retrovirus, papillomavirus, FeLV, FIV, Helicobacter, lymphoma, parasitic

## Abstract

**Simple Summary:**

Increasingly, cancers are being linked to infections with viruses, bacteria, and parasites in human medicine. This review summarises the current literature regarding neoplasia occurring in association with infectious diseases in domestic cats. To date, most studies have focused on the role of viruses, especially feline leukaemia virus and feline immunodeficiency virus in association with lymphoma, or the role of papillomavirus in cutaneous and oral neoplasms in cats. Recently, there has been a focus on a potential role of mouse mammary tumour virus in feline mammary carcinoma and lymphoma and studies assessing the potential role of gammaherpes virus and hepadnaviruses in feline neoplasia. Additionally, there has been some focus on potential bacterial and parasitic associations with neoplasia; including reports assessing potential associations between *Helicobacter* species and gastrointestinal neoplasms, and case reports of neoplasia in association with *Platynosomum fastosum* and *Opisthorchis viverrini.*

**Abstract:**

In recent years, growing attention has been paid to the influence/role of infectious diseases in tumour development and progression. Investigations have demonstrated that some infectious organisms can have a direct role in the development of neoplasia, whereas others can predispose to neoplasia by alterations in the immune response, or by creating a pro-inflammatory environment. Feline leukaemia virus was one of the first infectious agents recognised as an oncogenic organism, and along with feline immunodeficiency virus has received the most attention. Since the discovery of this retrovirus, several other organisms have been associated with neoplastic processes in cats, these include gammaherpes virus, mouse mammary tumour virus, papillomaviruses, hepadnavirus, *Helicobacter* species, and the parasitic infections *Platynosomum fastosum* and *Opisthorchis viverrini*. This review summarises the findings to date.

## 1. Introduction

In humans, cancer is typically considered a non-communicable disease. However, it is known that infectious agents can contribute significantly to the incidence of some cancers [1]. Indeed, the World Health Organization’s International Agency for Research on Cancer (IARC), lists the following biological agents as known carcinogens: *Helicobacter pylori* (*H. pylori*), Epstein–Barr virus (EBV), human herpes virus 8 (HHV8) (also known as Kaposi’s sarcoma human virus), human papillomaviruses (HPVs), human immunodeficiency virus 1 (HIV-1), hepatitis B and C (HBV, HCV), human T-cell lymphotrophic virus 1 (HTLV-1), Merkel cell polyomavirus (MCV) [2], and liver flukes: *Opisthorchis viverrini* and *Clonorchis sinensis* and *Schistosoma haematobium* (*S haematobium*) [3]. In addition, there are multiple other agents which have been implicated in various forms of neoplasia, with new evidence emerging continuously. Currently, it is estimated that globally 15% of human neoplasms are caused by infectious agents, with up to 30% reported in developing countries [4,5]. Particular regions in Africa, such as Kampala in Uganda, show an even higher incidence, with up to 50% of incident cancers being linked to infectious agents [6]. The association between infection and neoplasia varies depending on the malignancy; for example, 100% of cervical cancers are attributed to HPV infection, whereas (in specific geographic areas) up to 0.4% of liver cancer may be attributed to liver fluke [3].

Reports of infections causing cancer date back to 1911, when Peyton Rous first discovered a transmissible agent which could be isolated from the tumour cells of chickens; this would later become known as Rous sarcoma virus [7]. In the same year, Ferguson reported a high prevalence of neoplasia within the urinary bladder of men in Cairo and suggested that this was linked to *Schistosoma* parasites [8]. In 1936, John Bittner discovered a ‘milk factor’ that could be transmitted lactogenically from mice with mammary tumours and demonstrated that the offspring would develop tumours once mature [9]. In 1949, Graff et al. determined that the ‘milk factor’ described by Bittner was a virus, which could induce mammary tumours when it was injected into the peritoneal cavity, initially named mouse tumour virus, and later, mouse mammary tumour virus (MMTV) [10]. The next advances did not come until 1964, when the Epstein–Barr virus (EBV) was first cultured from lymphoblasts in Burkitt’s lymphoma [11]; the same year, William Jarrett first isolated the feline leukaemia virus (FeLV) and identified it as a cause of leukaemia and lymphoma in domestic cats [12]. The discovery of this pathogenic gamma-retrovirus buoyed investigations into the mechanisms of retroviral-induced cancers and oncogenes. Shortly after, the structure of the mouse mammary tumour virus was elucidated, identifying it as a beta-retrovirus [13]. Following on from this work came the discovery of the first human retrovirus, HTLV-1, as a cause of adult T-cell leukaemia/lymphoma [14,15]. With the discovery that retroviral infections could contribute to the development of neoplasia there came a great deal of research into other potential infectious causes of neoplasia.

Malignant transformation due to infectious disease can occur via two broad mechanisms. The agent can act as a direct carcinogen altering the expression of oncogenes; thereby, leading to the production of oncoproteins. These oncoproteins can then interact with cellular proteins, and ultimately lead to mutagenesis by the disruption of cell cycle check-points, inhibition of apoptosis, and enhancement of cell immortalisation. In feline medicine, there have been several viruses which demonstrate direct mutagenesis, these include FeLV and MMTV. Alternatively, neoplastic transformation can be driven via indirect mechanisms; these include induction of chronic inflammation, which, in turn, results in the production of inflammatory mediators, and the production of reactive oxygen species, which have direct mutagenic effects and promote tumour neovascularisation. Inflammation-induced neoplasms have been associated with *Helicobacter* organisms and, potentially, *Opisthorchis* infections. Furthermore, immune suppression induced by viruses such as feline immunodeficiency virus (FIV), not only predispose to infection with other agents, it is also thought to alter the immune surveillance, and with this, the ability to remove neoplastic cells by the host.

As the role of various different infections in neoplastic transformation has become better understood, there have been advances made in the prevention of tumours, and in potential therapies for various neoplasms. To date, these advances have predominantly been in the human field, but increasing our understanding of the role of infectious organisms in feline oncological disorders can equally help us to advance our understanding of the pathogenesis of feline oncological disorders and with this, help elucidate potential preventative or therapeutic targets to decrease the incidence of neoplasia in this species.

## 2. Viral Infections Associated with Neoplasia in the Cat

As the first infectious organisms associated with neoplasia, viruses, especially retroviruses, have received the most attention from researchers. In addition to the role that these infections play in tumour development, the use of retroviral infection to alter the host DNA and treat or prevent neoplasia is an area of great interest. Of the feline viral infections, the role of FeLV has been studied most extensively.

### 2.1. Feline Leukaemia Virus (FeLV)

Since its discovery in 1964, FeLV has been reported globally [16]. It is recognised that progressive infection with FeLV has been shown to increase the risk of lymphoma development by 60 times and is associated with the development of other forms of neoplasia [17,18]. At its peak, it was estimated that approximately one-third of all cancer related deaths in cats were attributable to FeLV infection [19]. However, today the prevalence of FeLV infection is markedly decreased [20]. Whilst this would be expected to have led to a significant decrease in the incidence of feline lymphoma, there is conflicting literature in support of this theory. In a 2005 study by Louwerens et al., which assessed the prevalence of lymphoma cases presenting to a veterinary teaching hospital between 1983 and 2003, there was an increased prevalence of lymphoma within their referral population [21]. However, a recent study assessing 562,446 cats from general practices in the U.K., reported an incidence of 32/100,000 (CI 26-35/100,000) [22]. This was slightly lower than the incidence reported by Dorn et al. in a study from the U.S.A. in 1968 [23]. Likewise, a German study documented a reduction in lymphoma cases in cats with progressive FeLV infection from 59% (years surveyed, 1980–1995) to 20% (years surveyed, 1996–1999) [24,25].

**Figure 1:**
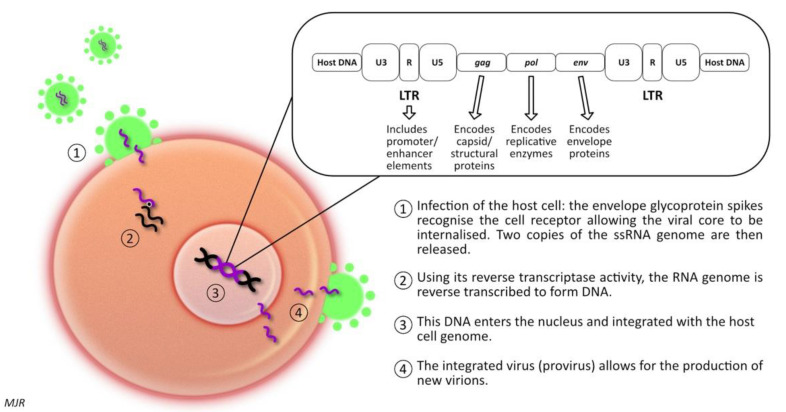
Schematic representation of FeLV integration within a host cell. The viral genome is depicted at the top of this image. The three genes (*gag*, *pol*, and *env*) within this simple retrovirus are flanked by long terminal repeats (LTRs) comprised of U3, R, and US regions. FeLV serves as an example of retroviral integration into the host DNA, with similar mechanisms employed by both FIV and MMTV.

When considering the oncogenic potential of FeLV infection, it is useful to consider the structure, function, and mechanisms of infection. FeLV is an enveloped single-stranded RNA virus with a genome containing three genes: the group specific antigen (*gag*) gene codes for the structural proteins of the virus; the polymerase (*pol*) gene codes for reverse transcriptase, protease, and integrase; and the envelope (*env*) gene codes for the envelope surface glycoprotein gp70 and transmembrane protein p15 [15,26] (Figure 1). In order to infect the host cell, the envelope glycoprotein spikes first recognise the cell receptor, allowing the viral core to be internalised [16]. Two copies of the single-stranded RNA (ssRNA) genome are then released, and using its reverse transcriptase activity, the RNA genome is reverse-transcribed to form DNA [15]. This enters the nucleus and is integrated into the host’s cell genome [27]. Like all retroviruses, the FeLV genome is flanked by long terminal repeats (LTRs) [16]. The integrated virus, known as provirus, then forms the template for production of new virions [15].

The term FeLV refers to a group of closely related viral subgroups, namely, exogenous FeLV-A, -B, -C, -D, -E, and -T, and endogenous FeLV (enFeLV), and endogenous retrovirus domestic cat (ERV-DC) [28,29,30,31,32,33,34,35,36,37,38,39,40,41,42]. Endogenous viruses form when proviral elements integrate into host germ cells, becoming a heritable part of the host genome [26]. During endogenisation, mutations frequently occur as reverse transcription is error-prone, having no proof-reading function [26]. Typically, an endogenised virus is incapable of producing infection, and may provide some protection against exogenous infection [26,43,44]. However, when enFeLVs recombine with exogenous FeLVs, notably in the *env* region, the biological activity and pathogenicity is altered [16].

The outcome of FeLV infection is highly variable, being influenced by the immune status and age of the cat, the pathogenicity of the virus, infection pressure, and virus concentration [20,45]. Infection most commonly occurs via the oronasal route, after which the virus replicates in local lymphoid tissues [20]. In many cats, replication is halted by cell mediated immunity and the virus is completely eliminated from the body [20]. In these cases, the virus never spreads systemically, and therefore, they do not react positively to antigen testing. These cats are known as “regressor cats”; they have high levels of neutralising antibodies and build a high level of immunity protecting them against viral challenge for many years [19,20]. However, if the immune response is not adequate, replicating FeLV spreads within mononuclear cells; this initial viraemic period may cause clinical signs of lethargy, pyrexia, and/or lymphadenopathy [19,20]. The virus then spreads to target organs which include the spleen, thymus, lymph nodes, and salivary glands. These cats secrete virus in their saliva and are infectious to other cats [19,20]; generally, this viraemia is transient, lasting only a few weeks before the majority of cats will clear the infection. These cats are termed “transiently viraemic” and will develop effective immunity protecting them against new exposures to virus [19,20].

In some cases, the infection is not cleared and the bone marrow will become involved, typically within 3 weeks of infection [19,20]. As a result, affected haematopoietic precursor cells produce infected granulocytes and platelets, which are released into the circulation, at which time there is a high level of viraemia within lymphoid organs and salivary glands also infected [45]. A percentage of these cats will go on to clear the viraemia, but do not completely eliminate the virus from the bone marrow; these cats are “latently infected” [19,20]. Cats which have latent infections can reactivate either spontaneously or when the immune system is suppressed (i.e., in pregnant cats or those receiving high doses of corticosteroids) [46,47]. Latently infected cats have the viral genome integrated into their cellular chromosomal DNA, which is replicated and passed onto daughter cells. However, the proviral DNA is not translated in proteins, so no infectious viral particles are produced [20]. The remaining cats remain “persistently infected”. It is this last group that the majority of FeLV associated mutagenesis has focused upon. Infection with FeLV likely has an indirect effect on lymphomagenesis by leading to immune suppression [16]. However, direct mechanisms are also thought to play a significant role in tumour formation.

As a simple retrovirus, the FeLV genome encodes only the genes required for its structure and replication, and therefore, no genes which directly confer malignancy [48]. One mechanism by which retroviruses can induce neoplasia is via transduction, whereby the virus acquires a copy of the oncogene during replication; this allows the oncogenic sequence to be transduced along with the virus (removing the gene from its normal transcription unit) [16,48,49]. As oncogenes encode a variety of products, including growth factors, growth factor receptors, and protein kinases, upregulation of these genes favours cell proliferation. This occurs in FeLV-induced multicentric fibrosarcoma; where feline sarcoma viruses (FeFSV) form via transduction of host cellular oncogenes, such as *fes, fms, fgr, abi*, and *kit* into FeLV-A [50,51]. FeFSV is an oncogenic virus, capable of causing polyclonal malignancy with multifocal tumours arising simultaneously after a short incubation period [50].

The second mechanism by which FeLV can induce neoplasia is via integration into the cellular genome. Gene expression in retroviruses is directed by the LTRs. The LTR of FeLV has three components, the U3, R, and U5 regions [49]. Of these, it is the U3 region which contains transcriptional promoter and enhancer sequences [52]. These can act on adjacent viral genes, or on cellular genes near to the site of proviral integration; for example, the U3 region has been demonstrated to transactivate cancer-related signalling pathways through the production of a non-coding 104 base RNA transcript that activates NF-KB [53]. If the retrovirus is integrated into the host cell genome near to, for example, a proto-oncogene, such as *myc*, transcription of that gene can be upregulated [54,55,56,57,58,59,60]. This may be mediated by the LTR’s as these contain promoter and enhancer functions; thereby, possessing the potential to regulate the transcription of adjacent cellular genes in the appropriate target cell [52]. Tandem repeats of enhancer motifs in the U3 region have been found within FeLV provirus integrated in the genomes of lymphoid neoplasms and leukaemias [16]. This suggests that the enhanced expression of the genes adjacent to the integrated proviral genome is associated with oncogenesis [61]. Additionally, integration into a tumour suppressor gene can disrupt cellular function, and thereby, the cell can acquire a growth advantage. To date, common integration sites (CISs) for the development of FeLV-associated lymphoma have been identified in six loci, namely: *c-myc, flvi-1, flvi-2, fit-1, pim-1*, and *flit-1* [57,58,62,63,64,65]. Of these, *c-myc* has been recognised as a proto-oncogene, coding for a nuclear phosphoprotein belonging to a family of transcription factors. In addition, *flvi-2, pim-1,* and *fit-1* have been identified as collaborators. More recently, it was discovered that insertion at the *flit-1* locus (which is interrupted in 20% of T-cell thymic lymphoma cases) is associated with the overexpression of activin-A receptor. Activin-A is a multifunctional cytokine, regulating cell growth and differentiation [48,49,66,67]. Insertion at the *flvi-1* locus is associated with the development of multicentric non-T-cell, non-B-cell lymphoma. Whilst the coding capacity of *flvi-1* is unknown, it is thought to encode an oncogene, the activity of which is altered by the adjacent integration of the proviral LTR [61,65].

It has been suggested that latent or regressive FeLV infection may contribute to lymphomagenesis [16]. In these instances, proviral integration could be responsible for the initiation of the neoplastic process. Early studies demonstrated antigen-negative, antibody-positive cats were at a greater risk of lymphoma than cats that had never been exposed to the virus [68,69]. In addition, some cases have alluded to a potential oncogenic role in regressive infection, with *myc* having been identified in T-cell lymphoma from an antigen-negative, antibody-positive cat [70,71]. However, when investigators have looked for provirus to investigate the outcomes in regressive FeLV infection, there are discordant results; some have suggested this is rare [72,73,74], whilst others have suggested as many as 20-60% of lymphomas from antigen-negative cats are provirus positive [75,76,77].

Recently, the potential role of enFeLV in lymphomagenesis has been investigated [43,44,78,79]. It has been shown that the enFeLV copy number is lower in cases with exogenous FeLV infection [43,44,79] and associated with a better disease outcome. In one study, females demonstrated a lower enFeLV copy number and were more likely to have progressive FeLV and FeLV-B infection than males. In a second study assessing the expression of enFeLV genes in cats with lymphoma, it was found that the expression of enFeLV is decreased in the lymphoma cases compared to control cats [80]. However, the significance of alterations in the enFeLV copy number and the role in the mutagenesis remains unclear.

### 2.2. Feline Immunodeficiency Virus (FIV)

Feline immunodeficiency virus (FIV) is a retrovirus of the lentivirus genus, first described in 1986 [81]. Like FeLV, FIV infection leads to the integration of proviral DNA into the host cell DNA; thereby, influencing host cell function. There are six different subtypes recognised, labelled A to F [82]. However, infection with one subtype does not protect against infection with a second subtype (superinfection) [82]. It has been demonstrated that in superinfected cats, the exchange of gene segments encoding the *env* protein from different subtypes can occur [82].

The prevalence of FIV infection varies widely among different geographic locations, with pockets of high prevalence of up to 47% in some groups of feral cats, compared to that of 2–5% as reported in some studies of healthy, owned cats [83]. Generally, seroprevalence is higher in male cats, and adult cats are more likely to be infected than young cats [84]. This pattern arises because of the mode of transmission; in natural circumstances, FIV is transmitted primarily via inoculation of the virus present in saliva or blood (i.e., cat fight wounds) [83]. Experimentally, the virus can be transmitted in utero, or postpartum via milk or via transmucosal (oral, intrarectal, or intravaginal) inoculation. However, there is no evidence that these routes of infection play an important role in the natural transmission of disease [82,85].

FIV-infected cats are five to six times more likely to develop lymphoid neoplasia than uninfected cats [18]. Concomitant infection with FIV and FeLV increases the likelihood of lymphoma development by nearly 80-fold [18]. FIV-associated lymphomas are predominantly high-grade B cell tumours arising at extra-nodal locations [86]. Nevertheless, T-cell and non-B, non-T-cell lymphomas have been described in FIV infected cats, as have various other forms of neoplasia, including mast cell tumour, fibrosarcoma, squamous cell carcinoma, and mammary carcinoma [87,88,89,90,91,92,93,94,95,96,97,98,99,100,101,102]. Unlike FeLV, it appears that direct oncogenesis by FIV is rare [88,91]. When proviral integration occurs before transformation, the provirus within the tumour DNA is monoclonal. In the majority of FIV-positive lymphoma tissues, clonal integration has not been identified [87,88,91,100]. Occasionally, direct integration does appear to be present [92,103,104]. In one experimentally infected cat, FIV sequences were detected in DNA isolated from a high grade-B-cell lymphoma [103]. Additional analysis demonstrated that the site of FIV integration was on a conserved gene in feline chromosome B3, which resulted in a promoter insertion and truncation of this gene [104]. The orthologous human gene (*FLJ12973*), located on chromosome 15q15, is frequently the site of deletions in ovarian and colorectal cancers. This integration location suggests that the FIV insertion may result in a loss of function of the affected gene [104]. A second study demonstrated FIV proviral sequences in 2 of 14 biopsies from naturally occurring lymphoma cases [92]. Interestingly, direct lymphomagenesis by HIV is also uncommon [105,106]. It has been suggested that this may be due to integration site preference, weak enhancer/promoter sequences, refractory target cells, and/or viral cytotoxicity [107].

In the majority of cases, it appears that FIV induces neoplasia via indirect mechanisms. It is recognised that there is an increased risk of neoplasia in feline renal transplant patients receiving cyclosporine [108,109]; it is thought that immune dysfunction, impaired surveillance and the removal of neoplastic cells, may play a role in FIV-induction of neoplasia [86]. The observation that cases with multiple neoplasms, for example, spinal lymphoma and concurrent disseminated mastocytoma in an FIV positive cat [110], supports the theory that virus-related immunosuppression may be a contributing factor in the development of malignancies.

In early FIV infection, B cell proliferation is increased, leading to a large number of lymphocytes [86]. During this period there is follicular hyperplasia, and expansion of B-cell regions, consistently resulting in a pre-neoplastic phenotype. This rapid proliferation may increase the likelihood of malignant transformation [100,111,112]. In a study assessing the immune alterations in two cats that developed FIV-associated lymphoma, increased lymphocyte blastogenesis and increased IL-1 and IL-6 (which stimulate B-cell proliferation and differentiation) was demonstrated [99]. In addition, these cats showed a reduced CD4-lymphocyte count; a finding which is a recognised risk factor for HIV-associated lymphoma [16,99]. Recently, multiple T-cell lymphomas were identified at post mortem in a case of terminal FIV. In that case, which was thought to have arisen from indirect mutagenesis, the proviral LTR was amplified and sequenced from multiple anatomical sites. The authors identified a novel activated phorbol myristate acetate (PMA) response element with the U3 region, the role of which remains undermined [113].

### 2.3. FIV & Gammaherpesviruses

Herpesviruses are a major cause of cancers in immunodeficient humans [114]. Some forms of herpesvirus encode several oncogenic proteins, which have been shown to inhibit cellular apoptosis and promote cell survival [86]. HIV-associated neoplasms are frequently associated with co-infection with gammaherpesvirus [114,115]. EBV, a human herpesvirus-4, can be responsible for infectious mononucleosis (glandular fever) [114]; although, typically, infection is asymptomatic if individuals are infected during childhood or in early adulthood. However, in immunocompromised humans (post-transplant or HIV/AIDS patients), EBV is linked to the formation of Burkitt’s and Hodgkin lymphomas [114]. Whilst EBV (or Epstein–Barr like virus) and a bovine herpesvirus-4 (BHV) have been reported in cats [116,117], follow-up studies have not been able to identify BHV, and to date, there are no reports of neoplasia associated with these infections [118].

Kaposi’s sarcoma is a human neoplasm, which occurs in association with HIV and HHV-8 infection [115]. Recognition of this association prompted investigations to identify a similar affiliation in feline medicine. In 2014, a feline gammaherpesvirus, Felis catus gammaherpesvirus 1 (FcaGHV1) was first detected [119,120]. Infection with FcaGHV1 has been reported in Australia, U.S.A., U.K., Europe, Singapore, Japan, and Brazil, with 9.6–23.6% of cats demonstrating viral DNA within their blood [119,120,121,122,123,124,125]. Male cats are generally at a higher risk of FcaGHV1 than females [79,121]. The virus is spread by oropharyngeal shedding, leading to the suggestion that, like FIV, it may be spread by biting [126]. In one study assessing over 200 cats, positive FcaGHV1 status was associated with a greater likelihood of being classified as ‘sick or unhealthy’ by the examining veterinarian [119]. The levels of FcaGHV1 DNA within the blood of FIV-positive cats, is higher than in age matched controls [119,121]. However, studies assessing a potential link between concurrent FIV and FcaGHV1 infections and lymphoma are sparse. One recent study did not find an association between FcaGHV1 and the development of high-grade lymphoma, but did report a shortened survival time in co-infected cats [124]. A second study, using in situ hybridisation, demonstrated FcaGHV1 within the malignant lymphocytes in 1/23 cases of FIV-associated lymphoma [127]. In a series of four cats with rapidly progressive FIV-associated lymphoma, ‘gammaherpesvirus’ was not identified [112].

### 2.4. Mouse Mammary Tumour Virus (MMTV)

MMTV is an oncogenic retrovirus that induces mammary carcinoma in mice and for which, possible links to human breast cancer have been suggested [128]. Some studies have demonstrated MMTV-like sequences (with 95% homogeneity to MMTV) are highly expressed in human breast cancer, and viral particles produced in primary cell cultures derived from breast cancer are similar to those of MMTV [129,130]. Furthermore, epidemiological studies have demonstrated that the geographic variations in the incidence of human breast cancer match the natural range of certain species of mice, particularly *Mus domesticus* [131]. Despite this, the potential role of MMTV in human breast cancer remains controversial, as studies in some geographic areas have demonstrated no evidence of MMTV-like sequences. It has been hypothesised that these studies are from areas where *Mus domesticus* is not endemic, or other factors may be involved [128,131]. Indeed, several aetiological agents have been linked to human breast cancer, including EBV, certain forms of HPV, bovine leukaemia virus (BLV), and, most recently, the bacterial microbiome [130]. To date, none of these are classified as recognised causes of neoplasia by the IARC. Despite this, there is a large body of research which has assessed the role of MMTV in the induction of mammary carcinoma.

MMTV is a beta-retrovirus, composed of prominent surface spikes with an eccentric condensed core. The genome codes for both structural and non-structural proteins, and therefore, MMTV is classified as a complex retrovirus [128,131]. The transcript codes for *Gag, Pol, pro-dUTPase* (*DUT*) protein and *Env,* flanked by LTRs [128]. These LTRs are exceptionally long, and encode for two additional genes: *sag* (a viral accessory protein that functions as a superantigen) and *rem* (which encodes an RNA export protein) [128]. MMTV replicates efficiently in the mammary alveolar epithelial cells, with increased expression observed during lactation due to the release of steroid hormones [128]. In mice, lactogenic transmission results in the virus infecting the dendritic cells and B lymphocytes of the Peyer’s patches. The *sag* antigen is then presented to CD4+ T-lymphocytes. This protein triggers viral replication and amplification of T-lymphocytes, which act as a carrier to transfer the virus from the gut to the mammary tissue. MMTV infection does not immediately trigger neoplastic transformation, even once proviral DNA is inserted in the cell DNA [128]. The insertion results in proto-oncogene deregulation (much like infection with FeLV). To date, rearrangements in several cellular gene families (*wnt, fgf, notch4/int3, rspo* and the gene encoding the p48 component of eukaryotic translation initiation factor-3 (*elF-3p48)*) have been demonstrated with MMTV infection [128,129]. It is hypothesised that these insertions are responsible for neoplastic transformation. However, indirect mechanisms for MMTV tumorigenesis have also been implicated [132]. It has been suggested that MMTV may activate an immunoreceptor tyrosine-based mechanism that suppresses apoptosis [132]. Alternatively, it has been hypothesised that MMTV infection could lead to the activation of other infections, such as EBV or HPV, which have also been implicated in the development of mammary neoplasia [132].

Infection of feline kidney cells with MMTV was first demonstrated 1976 [133,134]. However, it was only in 2005 that viral sequences similar to MMTV (>90%) were first isolated from feline tissues (thymus and spleen) [135]. In 2010, Hsu et al. first isolated MMTV-like nucleotide sequences from two out of nine (22.22%) feline mammary carcinomas [131]. In that study, no MMTV-like sequences were identified in benign mammary lesions (n = 3). However, MMTV-like sequences were identified in one of three cats with normal mammary tissue. Following on from this work, Civita et al. demonstrated MMTV-like *env* sequences in 6/86 (7%) of feline mammary carcinomas [136]. In that study MMTV-like *env* sequences were not isolated from normal feline mammary tissue (n=6). The authors of that study highlighted the presence of MMTV-like p14 protein (the signalling peptide of the MMTV *env*) localised within the cytoplasm and, occasionally, the nucleus of infected cells. In a follow-up study, Parisi et al. detected MMTV-like virus in 3 of 24 (12.5%) feline mammary carcinomas [132]. Additionally, that group of researchers have recently amplified MMTV *env-*like sequences and p14 antigen in 5/53 feline lymphoma samples [137]. The positive samples in that study were comprised of a mixture of gastrointestinal (n = 3) and nasal lymphoma (n = 2) and included a mixture of B- and T-cell lymphomas. Whilst these studies have assessed the viral sequences and potential tumour correlations, to date, none have been identified; highlighting the need for further investigations in this area.

### 2.5. Papillomaviruses

One of the strongest associations between an infectious agent and neoplasia in humans is the role of papillomaviruses in cervical carcinoma [2]. There have been more than 300 papillomaviruses identified and sequenced [138]. With the exception of some delta-papillomaviruses, papillomaviruses demonstrate genotype-specific host-restriction, with preference for distinct anatomic sites [138]. These double stranded circular DNA viruses infect epithelial basal cells; in order for infection to manifest, damage to the overlying epithelial layers must occur [139,140]. The life cycle of the virus is dependent on the progression of cells from the basal to the superficial cell layers [139]. Virion assembly only occurs as the cell nears the epithelial surface, with viral particles being released after the dead epithelial cell has sloughed and degraded [141]. Therefore, papillomaviruses are dependent on stimulating cell division within normally post-mitotic keratinocytes [142].

The viral genome generally contains one regulatory region, the upstream regulatory region (URR) or long control region (LCR) [138]. This contains transcription factor-binding sites, the replication origin and two groups of open-reading frames (ORFs), which are either E (early) or L (late) [138]. The ‘core’ genes include the E1 and E2 proteins and the L1 and L2 proteins. E1 encodes a virus-specific DNA helicase [143], whereas E2 is involved in viral transcription, replication, and genome partitioning [143]. L1 encodes the structural protein in the virus capsid, and L2 the minor capsid protein, which binds to the circular viral DNA to facilitate optimal genome encapsidation [143]. When present, E4, has some characteristics of a core protein [138]. It binds to cytokeratin filaments, disrupting their structure, and therefore plays a role in virus escape from the cornified epithelial layers [143]. The remaining viral genes E5, E6, and E7 are ‘accessory’ genes, which appear to facilitate replication in stratified epithelium [138]. In HPV infection, proteins E5, E6, and E7 play an important role in tumorigenesis. In some HPV infections, proteins E5, E6, and E7 have been shown to play an important role in oncogenesis; E6 and E7 cause functional inactivation of the main regulators of the cell cycle, tumour transformation suppressors, and the activation of telomerases, whilst E5 enables keratinocyte differentiation and immune evasion [143]. Recently it has been demonstrated that E6 isolated from a feline papillomavirus enhances p53 proteasomal degradation, suggesting that the role of the feline protein is similar to that of the HPV E6 protein [144].

Histologically, papillomavirus infection can induce cell changes, such as enlargement of the keratinocytes, with smudged blue-grey cytoplasm or a shrunken nucleus surrounded by a clear halo [141,145]. However, these changes occur when there is papillomavirus replication, and so they will only be present in recently developed lesions, and not in advanced lesions with well-differentiated cells [141]. To aid identification in advanced lesions, immunohistochemical markers were identified, utilising antibodies which detect the presence of L1 [139]. However, this viral capsid protein is only produced when the virus is replicating, which is rare in such lesions [141]. Therefore, in human diagnostic laboratories, it has become routine to stain for increased levels of p16 [141]. This is produced by the host cell when cell replication is stimulated by papillomavirus [141], with the p16^CDKN2A^ protein (abbreviated to p16) being increased even when the virus is not replicating [146]. Additionally, PCR or in situ hybridisation techniques can be employed to detect low levels of viral nucleic acid [147,148]. Where infection with papillomavirus is common, detection by PCR can be problematic, as the detection of low amounts of viral DNA does not prove causality [141]. In such circumstances in situ methods are preferable, as they allow the localisation of the viral nucleic acid. If papillomaviral nucleic acid is identified within the basal and suprabasilar layers, this suggests that the virus is likely to have played a role in lesion development [141].

There are currently six different feline papillomaviruses recognised; *Felis catus* papillomavirus types 1-6 (FcaPV 1-6) [141]. Cats are also dead-end hosts for the delta-papillomavirus *Bos taurus* papillomavirus-14 (BPV-14) [141]. The majority of cats are infected with FcaPV’s, but disease associated with infection is rare, suggesting that, in most circumstances, host defences inhibit viral replication [142]. Occasionally, papillomaviruses can induce a rapid (self-limited) increase in cell growth, resulting in a hyperplastic papilloma (wart) or a more modest increase in cell growth, leading to the development of a raised plaque [139]. The increased replication within cells can lead to neoplastic transformation [139]. There are various cutaneous lesions that have been associated with papillomavirus infection in cats. These include, cutaneous papilloma’s (warts), which are rare in the cat; viral plaques and Bowenoid in situ carcinoma (BISC); feline cutaneous squamous cell carcinoma (SCC); basal cell carcinoma (BCC), feline sarcoid, and Merkle cell carcinoma [149,150,151]. In addition, oral papilloma’s and oral squamous cell carcinoma (OSCC) have been linked to feline papillomavirus infection [141].

Feline viral plaques and BISC are thought to represent two extremes of the same disease process [142]. These lesions typically develop in middle to older aged cats [140]. They can be found anywhere on the body, but are most common around the head and neck. Viral plaques are small (less than 8mm) slightly raised, hairless lesions, whereas BISCs are larger, more markedly raised, ulcerated, and crusted lesions [141]. The lesions are considered pre-cancerous, but can progress to invasive squamous cell carcinoma in some cases [141]. Studies have demonstrated that both viral plaques and BISCs are frequently associated with FcaPV-2 infection [146,147,148,152,153,154,155,156]. The most common cause of viral plaques/BISC is FcaPV-2, which was identified in 48% of BISCs using immunohistochemistry [157]. More recently, these findings were corroborated by PCR in BISCs [154,158] and by fluorescence in situ hybridisation [148]. Occasionally, FcaPV-1, FcaPV-3, FcaPV-4, and FcaPV-5 have also been associated with the development of viral plaques/BISCs [147,153,156,159]. In the Devon Rex breed, BISCs have been reported to demonstrate a particularly aggressive form, with multiple lesions and rapid neoplastic transformation to SCC occurring. In one case report documenting aggressive behaviour of FcaPV-2 in a Devon Rex cat, increased p16 activity was identified as a potential cause [160]. A second study isolated FcaPV-2 from two Devon Rex BISC cases; one of these cases was also positive for E6/E7 gene expression [155], which are considered oncogenic proteins in HPV [138].

Cutaneous SCC in cats are most notably associated with UV-light exposure. However, there is evidence that FcaPVs may also play a role. Initially, it was noted that FcaPV-2 was identified more frequently in SCC than in non-SCC skin [158,161]. However, FcaPV-2 E6 and E7 RNA has also been identified in a proportion of SCCs, but not normal skin; more recently, the E6 and E7 proteins have been shown to influence neoplastic transformation [144,162,163]. FcaPVs have been detected in 76% of SCCs occurring in UV-protected (i.e., haired) areas, and 42% of SCCs from UV-exposed skin [164]. Furthermore, p16 has been identified in 84% of UV-protected SCC and 40% of UV-exposed SCC [146,164]. E6/E7 RNA has also been noted in 55% of UV-protected SCC cases and 0% of UV-exposed SCC’s, by in situ hybridisation [165]. Most frequently, FcaPV-2 has been identified, but in some cases FcaPV-3, FcaPV-4, or FcaPV-6 have also been detected [164,166,167,168].

BCCs are not common in cats [139]. However, it has been noted that they are often associated with adjacent BISC lesions, and therefore, it was suggested that FcaPVs may be linked to their occurrence. A cat with multiple BCCs that contained FcaPV-3 DNA has been reported, as has a case with a novel PV type [169,170]. These cases demonstrated classic papillomavirus-induced changes within the neoplastic cells. However, as so few feline papillomavirus-associated BCCs have been reported, the role of these infections in tumour formation remains undetermined.

Feline sarcoids are caused by proliferating mesenchymal cells. These lesions are thought to be caused by infection with BPV-14, which appears to be able to lead to cross-species infection. Only cats with contact to cattle are reported to be affected. There is no viral replication within the lesions, and no L1 immunostaining [171]. However, BPV-14 is consistently detected within feline sarcoids [172,173], but not in unaffected samples [174]. In situ hybridisation has demonstrated papillomavirus DNA within the neoplastic mesenchymal cells [171]. Together these support a causal role of BPV-14 in feline sarcoid formation.

Aside from the cutaneous manifestations, FcaPVs have been implicated in the development of oral lesions, oral papillomas, and OSCCs. Oral papillomas are rare in the cat; they tend to be restricted to the ventral surface of the tongue and are usually incidental findings [141]. In the few cases in which investigations have been performed, the lesions have demonstrated prominent papilloma-induced changes, and have stained positive for L1 or p16 [175,176]. Virus typing has only been performed in two cases, with FcaPV-1 being isolated in both of these [176].

OSCCs are aggressive, invasive neoplasms which are associated with short survival time, particularly when measures to achieve local control of the tumour are not pursued [141]. As papillomaviruses have been linked to human OSCCs and feline cutaneous SCCs, it has been hypothesised that FcaPVs may play a role in the formation of feline OSCCs. In two studies, assessing a total of 52 OSCCs, papillomavirus DNA was amplified from three tumours; there was no amplification from 20 non-neoplastic oral samples [177,178]. Of the three sequences amplified, two were found to be HPV, whereas the remaining one could not be sequenced. This could reflect infection or, equally, contamination. Overall, studies have produced highly variable results; both a study in New Zealand assessing a series of 30 OSCCs, and a study from Japan assessing seven OSCCs, failed to demonstrate the presence of FcaPV DNA [168,179]. However, some studies have detected FcaPVs, including another study from New Zealand, in which 36 OSCCs and 16 gingivitis cases were assessed; FcaPV-1 was identified in one case of OSCC and one of gingivitis [168,180]. In addition, a study from North America noted 1 of 20 OSCCs as being positive for FcaPV-3 [181]. However, a series from Italy demonstrated FcaPV-2 in 10 of 32 cats with OSCCs (although, in situ hybridisation was inconclusive) and 4 of 11 ulcerative gingivitis lesions; a series from Taiwan also demonstrated FcaPV-2 DNA in 11 of 19 OSCCs [162,166]. Collectively, these studies have not demonstrated increased p16 in cases with FcaPV amplification, or a greater presence of FcaPV DNA in neoplastic and non-neoplastic lesions [179]. Therefore, the role of FcaPV in OSCCs in cats remains undetermined.

Recently, it was reported that an in situ carcinoma of the gingiva and nictitating membrane of a cat demonstrated FcaPV-3 DNA and contained intense p16 staining, suggesting that PV infection may be implicated in the formation of in situ mucosal carcinoma in cats [182].

Merkle cell carcinoma (MCC) is a cutaneous neuroendocrine tumour, which is frequently observed in cats with either SCC or BCC. A recent study noted FcaPV-2 DNA in 20/21 MCCs, with in situ hybridisation revealing the presence of the E7 protein in 19/21 of these tumours [151]. Furthermore, p16 activity was increased and p53 decreased in 20/21 MCCs, suggesting that FcaPV-2 is associated with tumorigenesis in some MCC cases [151].

### 2.6. Hepadnavirus

In feline medicine, the majority of the research to date has focused on the roles of the retroviruses and papillomaviruses in neoplastic transformation. However, in humans, viral hepatopathies have long been established as a cause of hepatitis and hepatocellular carcinoma; approximately 35% of pathogen related cancers are attributed to hepatic neoplasia of viral origin [183]. The dominant viral causes of hepatic neoplasia in humans are hepatitis B and C [3]. Hepatitis B is caused by HBV; an enveloped, double stranded, DNA-based hepadnavirus, which can progressively incite hepatitis, and with this cirrhosis (an independent risk factor for HCC) [183]. However, whilst the exact mechanisms by which HBV infection results in hepatocellular carcinoma are still incompletely understood, it is recognised that HBV can lead to HCC with or without cirrhosis [183]. This is, in part, due to a direct effect of the virus. The HBV genome contains four genes (P, preC/C, S, and X). Gene P encodes polymerase; gene preC/C encodes HBcAg and hepatitis B envelope antigen; gene S encodes HBsAg; and gene X encodes a replication cofactor X. The X protein (HBx) derived from gene X plays an essential role in HBV pathogenesis and viral transcription, and has a central role in the oncogenic process [183].

Recently, a novel hepadnavirus, similar to HBV, has been identified in domestic cats (domestic cat hepadnavirus, DCH) [184]. Studies have demonstrated that 6.5–10.8% of pet cats are positive for DCH [184,185]. A retrospective study using PCR and in situ hybridisation techniques demonstrated that 43% of chronic hepatitis cases, and 28% of HCCs, were positive for DCH [186]. In comparison, no cats with cholangitis, biliary carcinoma, or normal liver, tested positive for DCH. Additionally, the histological features of the DCH-positive cases demonstrated histopathological features similar to those reported in humans with HBV [127]. This study provides some evidence that DCH may be involved in chronic hepatitis and hepatocellular carcinoma formation in cats, but further studies are needed to confirm these findings.

## 3. Bacterial Causes of Neoplasia

It has long been recognised that bacterial numbers are increased in tumour samples. However, little research had assessed a potential role for bacteria in neoplastic processes. One of the earliest reports documented increased numbers of *Escherichia coli (E. coli)* in samples of colonic neoplasia [187]. Following on from this, it was found that *Fusobacterium nucleatum* was increased in tumour tissues and correlated with lymph node metastasis, and that *Fusobacterium* was increased in colorectal cancer (CRC) [188,189]. In addition, *Helicobacter pylori (H. pylori)* was reported to be associated with pancreatic neoplasia [190]. It is now recognised that many forms of bacteria are associated with different neoplasms [191,192]. However, for many, the role that these organisms play, and their relative contribution to tumorigenesis, remains unknown. In cats, there is relatively scant research into the aetiopathogenic role of bacterial organisms in neoplasia. One recent study demonstrated increased numbers of mucosa-invasive bacteria in cats with large cell intestinal lymphoma (82%) than in cats with small cell lymphoma (18%) [193]. Furthermore, in that study, intravascular bacteria were observed only in cats with large cell lymphoma (29%), and serosal colonisation was more common in cats with large cell lymphoma (57%) than those with small cell lymphoma (11%). This study utilised fluorescent in situ hybridisation with a eubacterial probe, and therefore, the bacterial species were not deciphered [193].

### Helicobacter

A strong association between *H. pylori* and gastric cancer is recognised in humans, with 35–60% of gastric cancers being linked to *H. pylori* infection [194]. *H. pylori* produces several virulence-associated proteins, including Vac A, which has been shown to induce vacuolisation and apoptosis, and inhibit cell proliferation; Cag A, which induces proinflammatory cytokines in epithelial cells; Bab A, which leads to chronic inflammation and atrophic changes; Oip A, which contributes to proinflammatory cytokine (IL-8) expression; and Pic B, which induces IL-8 expression in gastric epithelial cells [195]. Of particular interest is Cag A, which has been linked to cell proliferation, inflammation, and anti-apoptosis behaviour via the induction of tumour necrosis factor-α (TNF-α), IL-1β, IL-6, IL-8, and IL-10 [195]. It has been suggested that *Helicobacter*-induced gastric cancer is due to increased cytokine release, inflammation, decreased apoptosis, and an increase in the mucosal proliferation [195].

MALT lymphoma is a specific form of gastric cancer associated with *Helicobacter* infection [196]. MALT lymphoma usually arises from marginal B cells, which are increased in gastritis caused by *Helicobacter* organisms [196]. These cells populate the mucosal associated lymphoid tissue, and displace/destroy the follicles within. In many instances, eradication of the *Helicobacter* organisms has been shown to be curative in the treatment of this low-grade MALT lymphoma [196].

**Figure 2:**
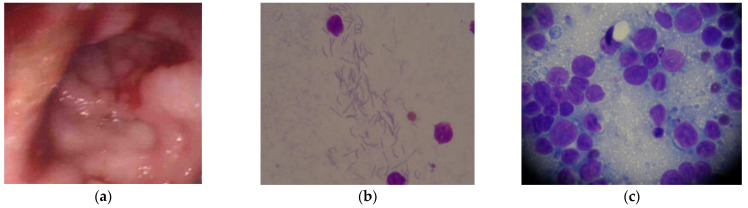
(**a**) Endoscopic appearance of the abnormal gastric mucosa; areas of mucosal proliferation and bleeding were seen giving the mucosa a ‘cobblestone’ appearance; (**b**) impression smear demonstrating multiple spiral-shaped organisms suggestive of Helicobacter-like organisms (×100); (**c**) impression smear demonstrating medium lymphocytes, with dense chromatin clumping, and multiple nucleoli (×400).

In cats, several enterohepatic *Helicobacter* species have been recognised. A study in 2001 demonstrated *Helicobacter* species in 17 of 45 cats [197]. Of these, nine were demonstrated as having *H. heilmannii,* four were positive for *H. felis,* three were positive for both *H. felis* and *H. heilmannii,* and seven for unclassified *Helicobacter* species [197]. There is a single report of *Helicobacter* organisms in cats with gastric lymphoma [196]. Of the cases with gastric lymphoma, 17 were identified with lymphoblastic lymphoma. In that study, histopathological samples from 47 cats with gastro-intestinal disease were assessed; 14 demonstrated gastritis, 31 lymphoma, and 2 were normal. It was found that ‘sick’ cats were more likely to host *Helicobacter* organisms. Furthermore, 13/17 cases of lymphoblastic lymphoma were positive for *H. heilmannii* by fluorescent in situ hybridisation [196]. Previously, one of the authors (KER) has diagnosed an 11-year-old, domestic cat with concurrent Helicobacter-like organisms and B-cell gastric lymphoma. That cat presented for a 1-month history of vomiting, weight loss, and decreased appetite. Investigations demonstrated a marginal increase in BUN, but no other abnormalities (FeLV and FIV tests were negative). Abdominal ultrasonography demonstrated regional thickening of the gastric wall (8.3 mm) at the pylorus. Endoscopy was performed (Figure 2a) and impression smears demonstrated multiple medium sized lymphocytes, as well as spiral-shaped, Helicobacter-like organisms (Figure 2b,c). Initially, the cat received a 14-day course of treatment for *Helicobacter* infection (amoxycillin-clavulanate, clarithromycin and ranitidine). Histopathology confirmed the diagnosis of B-cell lymphoma. At this time, all clinical signs had resolved. Treatment was commenced with a single dose of vincristine before the owner decided to not pursue further chemotherapy. A 1-month course of prednisolone, ranitidine and sucralphate was dispensed to provide palliative care. The cat was followed over the next 6 years; a repeat ultrasound performed 10-months after diagnosis demonstrated resolution of the gastric wall thickening. The cat was euthanised for presumed unrelated disease (chronic kidney disease).

*Helicobacter* organisms have been implicated in disease within the liver and intestine in cats. In the liver, organisms have occasionally been identified in cases of cholangiohepatitis or cholangitis [198]. However, in that study, organisms were identified with a similar frequency in normal tissue. Conversely, in a study assessing 55 intestinal carcinoma biopsies, *Helicobacter* organisms were identified in 56% of cases. In addition, the presence of *Helicobacter* organisms was significantly associated with large intestinal location (68%) and with mucinous adenocarcinoma (92%) [199].

## 4. Parasitic Infections

One of the first reports of neoplasia occurring in association with infection was the observation that bladder cancers were more prevalent in men with schistosomiasis [8]. Since this time, it has been demonstrated in humans that the helminth-associated diseases, schistosomiasis, opisthorchiasis, and clonorchiasis, are highly carcinogenic [200]. Furthermore, it has been shown that the protozoal organism, *Trypanosoma cruzi,* has a dual role of being both carcinogenic and anti-carcinogenic [200]. The malaria parasite, *Plasmodium falciparum,* is strongly associated with Burkitt lymphoma in endemic areas, but only when co-infection with EBV is present [200].

There are few reports of neoplasia associated with parasitic infection in the cat. One early report documented four cases of cholangiocarcinoma in cats parasitised by *Platynosomum fastosum (P. fastosum)* [201]. However, despite numerous reports of biliary tract infection by this trematode, there were no further reports of neoplasia until 2012, when post mortems of 3 of 11 cats infected with *P. fastosum* were reported to have cholangiocarcinoma [202]. In addition, there is a single case report of a cat with biliary cystadenoma associated with *Opisthorchis viverrini* infection [203], and, to date, there are no reports of *Opisthorchis felineus* leading to biliary neoplasia in the cat, despite reports in many other species [204,205,206].

## 5. Concluding Remarks

Infectious causes of neoplasia is a rapidly evolving area of interest in human medicine and there are many examples in veterinary medicine. Current research in feline medicine has focused predominantly on the retroviruses and FcaPVs, where a direct oncogenic role has been demonstrated in FeLV, FIV (occasionally), and some FcaPVs. The role of concurrent infections, such as FIV and FcaGHV-1, is currently being explored. Additionally, there is emerging evidence that other organisms, such as MMTV, hepadnavirus, *Helicobacter* infections, as well as other bacterial or parasitic infections, may be involved in neoplastic processes, either by direct or indirect mechanisms.

There is clear evidence that FeLV has a direct effect on the cell, inducing neoplastic transformation. However, the potential protective role enFeLV and the mechanisms by which LTRs influence mutagenesis is not clearly understood. A better understanding of these processes could lead to advances in our ability to protect from tumorigenesis. Despite these gaps in our knowledge, FeLV is the best understood mutagenic organism in the feline species. The precise mechanisms by which FIV and FcaGHV-1 initiate neoplastic transformation, and the role of co-infection with these viruses, remains unknown. Recently, there have been some advances in our understanding of MMTV in humans, but as yet, the role of this infection in feline neoplasms is unknown. Outside of the retroviral infections, FcaPVs have been most widely investigated, and both direct and indirect mechanisms of tumorigenesis have been identified. However, the role of FcaPVs in certain forms of neoplasia is a rapidly evolving area of interest. The recent discovery of hepadnavirus and a potential role of DCH in feline hepatic diseases is currently in its infancy. However, if infection mirrors disease in other species, there could be the potential to protect against some forms of hepatic neoplasia.

Documented evidence of bacterial or parasitic causes of neoplasia in the cat are sparse, with the only a few case reports detailing neoplasms in cats with bacterial or parasitic infections. Despite this, there is evidence of tumorigenesis occurring secondary to these organisms in other species. Considering these associations, it is important that veterinarians consider a potential role of infections in the development of neoplasms. Developing a better understanding of these processes, including the specific mechanisms by which different organisms influence the neoplastic process, could help in protecting against/treating various forms of neoplasia in the future.

## Data Availability

Not applicable.
